# 9-(7-Fluoro-4-oxo-4*H*-chromen-3-yl)-3,3,6,6-tetra­methyl-2,3,4,5,6,7,8,9-octa­hydro-1*H*-xanthene-1,8-dione

**DOI:** 10.1107/S1600536811051749

**Published:** 2011-12-07

**Authors:** Mohammad Asad, Chuan-Wei Oo, Hasnah Osman, Hoong-Kun Fun, Suhana Arshad

**Affiliations:** a School of Chemical Sciences, Universiti Sains Malaysia, 11800 USM, Penang, Malaysia; b X-ray Crystallography Unit, School of Physics, Universiti Sains Malaysia, 11800 USM, Penang, Malaysia

## Abstract

In the title compound, C_26_H_25_FO_5_, the terminal cyclo­hexane rings of the xanthene ring system adopt half-boat conformations. The 4*H*-chromene ring make a dihedral angle of 87.94 (5)° with the xanthene ring system and its carbonyl O atom lies above the xanthene O atom. In the crystal, mol­ecules are linked into ribbons propagating along the *a*-axis direction by C—H⋯O hydrogen bonds. Aromatic π–π stacking inter­actions [centroid–centroid distance = 3.7367 (12) Å] also occur.

## Related literature

For a related structure and background to the properties and applications of xanthene derivatives, see: Mehdi *et al.* (2011 ▶). For ring conformations, see: Cremer & Pople (1975 ▶). For reference bond-length data, see: Allen *et al.* (1987 ▶).
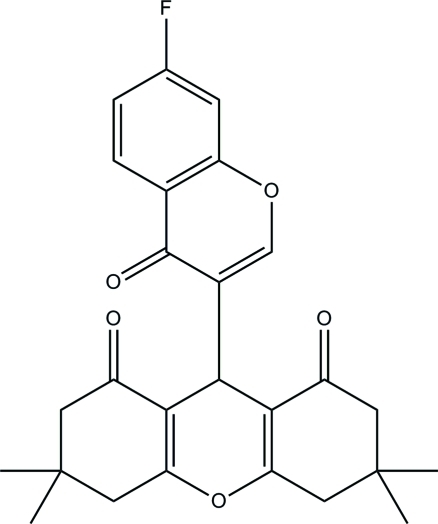


## Experimental

### 

#### Crystal data

C_26_H_25_FO_5_
*M* *_r_* = 436.46
Monoclinic, 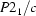

*a* = 6.9475 (8) Å
*b* = 18.596 (2) Å
*c* = 17.559 (2) Å
β = 93.658 (2)°
*V* = 2264.0 (5) Å^3^
*Z* = 4
Mo *K*α radiation
μ = 0.09 mm^−1^
*T* = 296 K
0.51 × 0.38 × 0.24 mm

#### Data collection

Bruker SMART APEXII DUO CCD diffractometer
Absorption correction: multi-scan (*SADABS*; Bruker, 2009 ▶) *T*_min_ = 0.954, *T*_max_ = 0.978
21143 measured reflections
6574 independent reflections
4315 reflections with *I* > 2σ(*I*)
*R*_int_ = 0.034

#### Refinement

*R*[*F* ^2^ > 2σ(*F* ^2^)] = 0.052
*wR*(*F* ^2^) = 0.150
*S* = 1.03
6574 reflections
293 parameters
H-atom parameters constrained
Δρ_max_ = 0.24 e Å^−3^
Δρ_min_ = −0.22 e Å^−3^

### 

Data collection: *APEX2* (Bruker, 2009 ▶); cell refinement: *SAINT* (Bruker, 2009 ▶); data reduction: *SAINT*; program(s) used to solve structure: *SHELXTL* (Sheldrick, 2008 ▶); program(s) used to refine structure: *SHELXTL*; molecular graphics: *SHELXTL*; software used to prepare material for publication: *SHELXTL* and *PLATON* (Spek, 2009 ▶).

## Supplementary Material

Crystal structure: contains datablock(s) global, I. DOI: 10.1107/S1600536811051749/hb6535sup1.cif

Structure factors: contains datablock(s) I. DOI: 10.1107/S1600536811051749/hb6535Isup2.hkl

Supplementary material file. DOI: 10.1107/S1600536811051749/hb6535Isup3.cml

Additional supplementary materials:  crystallographic information; 3D view; checkCIF report

## Figures and Tables

**Table 1:** Hydrogen-bond geometry (Å, °)

*D*—H⋯*A*	*D*—H	H⋯*A*	*D*⋯*A*	*D*—H⋯*A*
C15—H15*B*⋯O4^i^	0.97	2.44	3.3487 (19)	156
C18—H18*A*⋯O5^i^	0.97	2.45	3.3821 (19)	162

Symmetry code: (i) 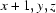.

**Fig. 1.**
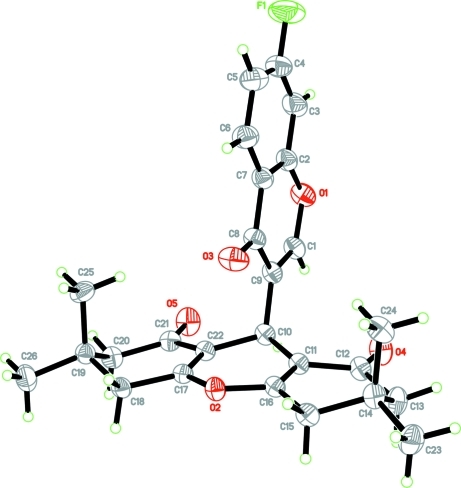
The molecular structure of the title compound with 30% probability displacement ellipsoids.

**Fig. 2.**
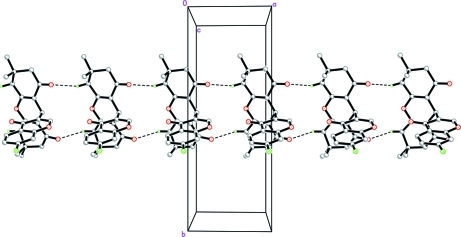
The crystal packing of the title compound. The H atoms not involved in the intermolecular interactions (dashed lines) have been omitted for clarity.

## supplementary crystallographic information

### Comment

As part of our ongoing studies of xanthene derivatives (Mehdi *et al.*, 2011), we now describe the synthesis and structure of the title compound, (I).

In the molecular structure, (Fig. 1), the terminal cyclohexane rings (C11–C16 & C17–C22) of the xanthene ring system adopt half boat conformations with puckering parameters Q= 0.4688 (18) Å, Θ= 124.2 (2)°, φ= 348.2 (3)° and Q= 0.4638 (17) Å, Θ= 54.8 (2)°, φ= 129.1 (2)° (Cremer & Pople, 1975), respectively. The 4*H*-chromene ring (O1/C1–C9) is approximately perpendicular to the xanthene ring system (O2/C10–C22) with dihedral angle of 87.94 (5)°. Bond lengths (Allen *et al.*, 1987) and angles are within normal ranges and comparable to a related structure (Mehdi *et al.*, 2011).

The crystal packing is shown in Fig. 2. The molecules are linked into ribbons along the *a* axis *via* intermolecular C15—H15B···O4 and C18—H18A···O5 hydrogen bonds (Table 1). π–π interactions of *Cg*1···*Cg*1 = 3.7367 (12) Å [*Cg*1 is the centroid of the benzene ring (C2–C7); symmetry code: -*x*, 1 - *y*, 2 - *z*] further stabilized the structure.

### Experimental

A mixture of 7-fluoro-3-formylchromone (2.60 mmol, 0.50 g) and dimedone (5.20 mmol, 0.73 g) in 30 ml methanol was stirred at room temperature overnight. The reaction progress was monitored by TLC. After the reaction was completed, the precipitate obtained was filtered, washed with methanol and dried. The isolated product was further purified by recrystallization from chloroform-methanol (1:1 *v*/*v*) to give the pure title compounds as colorless blocks in 92% yield.

### Refinement

All H atoms were positioned geometrically [C–H = 0.93–0.98 Å] and refined using a riding model with *U*_iso_(H) = 1.2 or 1.5 *U*_eq_(C). A rotating group model was applied to the methyl groups.

### Crystal data

**Table:**

C_26_H_25_FO_5_	*F*(000) = 920
*M**_r_* = 436.46	*D*_x_ = 1.281 Mg m^−^^3^
Monoclinic, *P*2_1_/*c*	Mo *K*α radiation, λ = 0.71073 Å
Hall symbol: -P 2ybc	Cell parameters from 5340 reflections
*a* = 6.9475 (8) Å	θ = 2.5–29.3°
*b* = 18.596 (2) Å	µ = 0.09 mm^−^^1^
*c* = 17.559 (2) Å	*T* = 296 K
β = 93.658 (2)°	Block, colourless
*V* = 2264.0 (5) Å^3^	0.51 × 0.38 × 0.24 mm
*Z* = 4	

### Data collection

**Table:**

Bruker SMART APEXII DUO CCD diffractometer	6574 independent reflections
Radiation source: fine-focus sealed tube	4315 reflections with *I* > 2σ(*I*)
graphite	*R*_int_ = 0.034
φ and ω scans	θ_max_ = 30.0°, θ_min_ = 1.6°
Absorption correction: multi-scan (*SADABS*; Bruker, 2009)	*h* = −9→9
*T*_min_ = 0.954, *T*_max_ = 0.978	*k* = −24→26
21143 measured reflections	*l* = −24→24

### Refinement

**Table:**

Refinement on *F*^2^	Primary atom site location: structure-invariant direct methods
Least-squares matrix: full	Secondary atom site location: difference Fourier map
*R*[*F*^2^ > 2σ(*F*^2^)] = 0.052	Hydrogen site location: inferred from neighbouring sites
*wR*(*F*^2^) = 0.150	H-atom parameters constrained
*S* = 1.03	*w* = 1/[σ^2^(*F*_o_^2^) + (0.0621*P*)^2^ + 0.5163*P*] where *P* = (*F*_o_^2^ + 2*F*_c_^2^)/3
6574 reflections	(Δ/σ)_max_ = 0.001
293 parameters	Δρ_max_ = 0.24 e Å^−^^3^
0 restraints	Δρ_min_ = −0.22 e Å^−^^3^

### Special details

**Table:**

Geometry. All e.s.d.'s (except the e.s.d. in the dihedral angle between two l.s. planes) are estimated using the full covariance matrix. The cell e.s.d.'s are taken into account individually in the estimation of e.s.d.'s in distances, angles and torsion angles; correlations between e.s.d.'s in cell parameters are only used when they are defined by crystal symmetry. An approximate (isotropic) treatment of cell e.s.d.'s is used for estimating e.s.d.'s involving l.s. planes.
Refinement. Refinement of *F*^2^ against ALL reflections. The weighted *R*-factor *wR* and goodness of fit *S* are based on *F*^2^, conventional *R*-factors *R* are based on *F*, with *F* set to zero for negative *F*^2^. The threshold expression of *F*^2^ > σ(*F*^2^) is used only for calculating *R*-factors(gt) *etc*. and is not relevant to the choice of reflections for refinement. *R*-factors based on *F*^2^ are statistically about twice as large as those based on *F*, and *R*- factors based on ALL data will be even larger.

### Fractional atomic coordinates and isotropic or equivalent isotropic displacement parameters (Å^2^)

**Table:**

	*x*	*y*	*z*	*U*_iso_*/*U*_eq_	
F1	0.0382 (3)	0.36000 (10)	1.08974 (8)	0.1100 (6)	
O1	−0.25037 (17)	0.46251 (7)	0.86767 (7)	0.0562 (3)	
O2	0.29558 (13)	0.54942 (6)	0.61824 (6)	0.0400 (2)	
O3	0.26966 (16)	0.48699 (8)	0.77520 (7)	0.0599 (4)	
O4	−0.21687 (16)	0.66244 (8)	0.73490 (8)	0.0624 (4)	
O5	−0.25611 (16)	0.40218 (7)	0.62320 (8)	0.0626 (4)	
C1	−0.2364 (2)	0.48941 (9)	0.79629 (10)	0.0467 (4)	
H1A	−0.3502	0.5028	0.7692	0.056*	
C2	−0.0864 (3)	0.43986 (9)	0.90721 (10)	0.0491 (4)	
C3	−0.1071 (3)	0.41034 (11)	0.97924 (11)	0.0654 (5)	
H3A	−0.2278	0.4060	0.9988	0.079*	
C4	0.0547 (4)	0.38826 (12)	1.01969 (12)	0.0713 (6)	
C5	0.2370 (4)	0.39369 (13)	0.99353 (12)	0.0735 (6)	
H5A	0.3447	0.3783	1.0233	0.088*	
C6	0.2552 (3)	0.42249 (11)	0.92207 (11)	0.0612 (5)	
H6A	0.3768	0.4260	0.9031	0.073*	
C7	0.0939 (2)	0.44652 (9)	0.87767 (9)	0.0462 (4)	
C8	0.1115 (2)	0.47825 (9)	0.80182 (9)	0.0438 (4)	
C9	−0.0704 (2)	0.49818 (9)	0.76172 (9)	0.0402 (3)	
C10	−0.07496 (19)	0.52939 (8)	0.68162 (8)	0.0384 (3)	
H10A	−0.2097	0.5382	0.6642	0.046*	
C11	0.03248 (19)	0.59992 (8)	0.68064 (8)	0.0379 (3)	
C12	−0.0564 (2)	0.66493 (9)	0.71072 (9)	0.0444 (4)	
C13	0.0533 (2)	0.73440 (10)	0.70679 (12)	0.0560 (4)	
H13A	0.0127	0.7664	0.7464	0.067*	
H13B	0.0203	0.7571	0.6579	0.067*	
C14	0.2729 (2)	0.72520 (9)	0.71637 (10)	0.0455 (4)	
C15	0.3311 (2)	0.67069 (9)	0.65689 (9)	0.0426 (3)	
H15A	0.3234	0.6934	0.6071	0.051*	
H15B	0.4642	0.6566	0.6685	0.051*	
C16	0.20817 (19)	0.60537 (8)	0.65378 (8)	0.0368 (3)	
C17	0.18772 (19)	0.48912 (8)	0.60097 (8)	0.0354 (3)	
C18	0.2912 (2)	0.43901 (8)	0.55154 (9)	0.0406 (3)	
H18A	0.4275	0.4393	0.5674	0.049*	
H18B	0.2773	0.4558	0.4992	0.049*	
C19	0.2145 (2)	0.36198 (9)	0.55528 (9)	0.0423 (3)	
C20	−0.0053 (2)	0.36473 (9)	0.54550 (10)	0.0474 (4)	
H20A	−0.0428	0.3803	0.4940	0.057*	
H20B	−0.0557	0.3166	0.5517	0.057*	
C21	−0.0958 (2)	0.41424 (9)	0.60100 (9)	0.0420 (3)	
C22	0.01150 (19)	0.47845 (8)	0.62625 (8)	0.0367 (3)	
C23	0.3742 (3)	0.79644 (10)	0.70260 (13)	0.0630 (5)	
H23A	0.3381	0.8132	0.6520	0.094*	
H23B	0.5113	0.7895	0.7080	0.094*	
H23C	0.3369	0.8313	0.7392	0.094*	
C24	0.3312 (3)	0.69813 (11)	0.79693 (10)	0.0565 (5)	
H24A	0.4685	0.6916	0.8022	0.085*	
H24B	0.2684	0.6531	0.8054	0.085*	
H24C	0.2934	0.7327	0.8337	0.085*	
C25	0.2778 (3)	0.32836 (10)	0.63219 (11)	0.0555 (4)	
H25A	0.2259	0.2806	0.6348	0.083*	
H25B	0.2313	0.3571	0.6726	0.083*	
H25C	0.4161	0.3262	0.6375	0.083*	
C26	0.2962 (3)	0.31780 (11)	0.49147 (12)	0.0645 (5)	
H26A	0.2458	0.2698	0.4925	0.097*	
H26B	0.4343	0.3163	0.4987	0.097*	
H26C	0.2600	0.3396	0.4431	0.097*	

### Atomic displacement parameters (Å^2^)

**Table:**

	*U*^11^	*U*^22^	*U*^33^	*U*^12^	*U*^13^	*U*^23^
F1	0.1401 (15)	0.1261 (13)	0.0658 (8)	0.0020 (11)	0.0231 (9)	0.0400 (8)
O1	0.0411 (6)	0.0750 (8)	0.0545 (7)	−0.0079 (6)	0.0193 (5)	0.0016 (6)
O2	0.0267 (5)	0.0454 (6)	0.0489 (6)	−0.0015 (4)	0.0099 (4)	−0.0043 (5)
O3	0.0291 (5)	0.0953 (10)	0.0562 (7)	0.0035 (6)	0.0084 (5)	0.0158 (7)
O4	0.0302 (6)	0.0743 (9)	0.0838 (9)	0.0068 (5)	0.0124 (6)	−0.0161 (7)
O5	0.0307 (6)	0.0703 (9)	0.0874 (10)	−0.0117 (5)	0.0089 (6)	−0.0105 (7)
C1	0.0328 (7)	0.0583 (10)	0.0502 (9)	−0.0036 (7)	0.0112 (6)	−0.0029 (7)
C2	0.0518 (9)	0.0494 (9)	0.0476 (9)	−0.0067 (7)	0.0141 (7)	−0.0031 (7)
C3	0.0758 (13)	0.0663 (13)	0.0569 (11)	−0.0111 (10)	0.0256 (10)	0.0029 (9)
C4	0.0988 (17)	0.0660 (13)	0.0505 (11)	−0.0014 (12)	0.0167 (11)	0.0118 (9)
C5	0.0844 (15)	0.0763 (15)	0.0595 (12)	0.0116 (12)	0.0021 (11)	0.0148 (10)
C6	0.0567 (11)	0.0704 (13)	0.0569 (11)	0.0086 (9)	0.0069 (8)	0.0093 (9)
C7	0.0450 (8)	0.0495 (9)	0.0449 (8)	−0.0012 (7)	0.0085 (7)	−0.0006 (7)
C8	0.0337 (7)	0.0547 (9)	0.0437 (8)	0.0001 (6)	0.0067 (6)	0.0002 (7)
C9	0.0281 (6)	0.0493 (9)	0.0441 (8)	−0.0018 (6)	0.0086 (6)	−0.0037 (7)
C10	0.0198 (6)	0.0507 (9)	0.0448 (8)	0.0012 (5)	0.0042 (5)	−0.0020 (6)
C11	0.0256 (6)	0.0478 (8)	0.0403 (7)	0.0026 (6)	0.0022 (5)	−0.0018 (6)
C12	0.0278 (7)	0.0563 (10)	0.0487 (9)	0.0072 (6)	−0.0007 (6)	−0.0057 (7)
C13	0.0418 (9)	0.0516 (10)	0.0745 (12)	0.0079 (7)	0.0023 (8)	−0.0099 (9)
C14	0.0379 (8)	0.0469 (9)	0.0517 (9)	−0.0029 (6)	0.0044 (6)	−0.0069 (7)
C15	0.0324 (7)	0.0503 (9)	0.0456 (8)	−0.0048 (6)	0.0070 (6)	−0.0011 (7)
C16	0.0278 (6)	0.0450 (8)	0.0377 (7)	0.0022 (6)	0.0028 (5)	−0.0009 (6)
C17	0.0280 (6)	0.0414 (8)	0.0367 (7)	0.0003 (5)	0.0019 (5)	0.0020 (6)
C18	0.0342 (7)	0.0466 (8)	0.0418 (8)	0.0029 (6)	0.0088 (6)	0.0014 (6)
C19	0.0376 (7)	0.0465 (9)	0.0430 (8)	0.0029 (6)	0.0054 (6)	0.0001 (7)
C20	0.0415 (8)	0.0520 (10)	0.0479 (9)	−0.0032 (7)	−0.0034 (7)	−0.0050 (7)
C21	0.0281 (7)	0.0509 (9)	0.0461 (8)	0.0004 (6)	−0.0031 (6)	0.0030 (7)
C22	0.0250 (6)	0.0462 (8)	0.0388 (7)	0.0018 (5)	0.0009 (5)	0.0010 (6)
C23	0.0614 (12)	0.0505 (11)	0.0772 (13)	−0.0082 (8)	0.0057 (10)	−0.0060 (9)
C24	0.0476 (10)	0.0734 (12)	0.0486 (9)	−0.0106 (8)	0.0038 (7)	−0.0075 (9)
C25	0.0487 (9)	0.0567 (11)	0.0611 (11)	0.0096 (8)	0.0034 (8)	0.0174 (9)
C26	0.0683 (12)	0.0607 (12)	0.0662 (12)	0.0054 (9)	0.0177 (10)	−0.0122 (9)

### Geometric parameters (Å, °)

**Table:**

F1—C4	1.349 (2)	C14—C23	1.527 (2)
O1—C1	1.359 (2)	C14—C15	1.528 (2)
O1—C2	1.363 (2)	C14—C24	1.532 (2)
O2—C17	1.3719 (18)	C15—C16	1.484 (2)
O2—C16	1.3744 (17)	C15—H15A	0.9700
O3—C8	1.2317 (18)	C15—H15B	0.9700
O4—C12	1.2192 (18)	C17—C22	1.3433 (18)
O5—C21	1.2243 (18)	C17—C18	1.489 (2)
C1—C9	1.3470 (19)	C18—C19	1.531 (2)
C1—H1A	0.9300	C18—H18A	0.9700
C2—C7	1.391 (2)	C18—H18B	0.9700
C2—C3	1.395 (3)	C19—C20	1.526 (2)
C3—C4	1.354 (3)	C19—C25	1.527 (2)
C3—H3A	0.9300	C19—C26	1.528 (2)
C4—C5	1.378 (3)	C20—C21	1.507 (2)
C5—C6	1.378 (3)	C20—H20A	0.9700
C5—H5A	0.9300	C20—H20B	0.9700
C6—C7	1.397 (3)	C21—C22	1.461 (2)
C6—H6A	0.9300	C23—H23A	0.9600
C7—C8	1.469 (2)	C23—H23B	0.9600
C8—C9	1.455 (2)	C23—H23C	0.9600
C9—C10	1.520 (2)	C24—H24A	0.9600
C10—C22	1.509 (2)	C24—H24B	0.9600
C10—C11	1.510 (2)	C24—H24C	0.9600
C10—H10A	0.9800	C25—H25A	0.9600
C11—C16	1.3402 (18)	C25—H25B	0.9600
C11—C12	1.471 (2)	C25—H25C	0.9600
C12—C13	1.503 (3)	C26—H26A	0.9600
C13—C14	1.534 (2)	C26—H26B	0.9600
C13—H13A	0.9700	C26—H26C	0.9600
C13—H13B	0.9700		
			
C1—O1—C2	118.51 (12)	C14—C15—H15B	109.0
C17—O2—C16	117.92 (10)	H15A—C15—H15B	107.8
C9—C1—O1	125.01 (16)	C11—C16—O2	122.77 (14)
C9—C1—H1A	117.5	C11—C16—C15	125.73 (14)
O1—C1—H1A	117.5	O2—C16—C15	111.49 (11)
O1—C2—C7	121.73 (15)	C22—C17—O2	122.97 (13)
O1—C2—C3	116.91 (16)	C22—C17—C18	125.75 (14)
C7—C2—C3	121.36 (18)	O2—C17—C18	111.29 (11)
C4—C3—C2	117.71 (19)	C17—C18—C19	112.21 (12)
C4—C3—H3A	121.1	C17—C18—H18A	109.2
C2—C3—H3A	121.1	C19—C18—H18A	109.2
F1—C4—C3	118.7 (2)	C17—C18—H18B	109.2
F1—C4—C5	117.8 (2)	C19—C18—H18B	109.2
C3—C4—C5	123.53 (19)	H18A—C18—H18B	107.9
C6—C5—C4	118.2 (2)	C20—C19—C25	110.06 (13)
C6—C5—H5A	120.9	C20—C19—C26	110.59 (15)
C4—C5—H5A	120.9	C25—C19—C26	109.19 (15)
C5—C6—C7	121.02 (19)	C20—C19—C18	108.20 (13)
C5—C6—H6A	119.5	C25—C19—C18	109.84 (14)
C7—C6—H6A	119.5	C26—C19—C18	108.95 (13)
C2—C7—C6	118.21 (16)	C21—C20—C19	113.78 (13)
C2—C7—C8	120.19 (15)	C21—C20—H20A	108.8
C6—C7—C8	121.60 (15)	C19—C20—H20A	108.8
O3—C8—C9	123.44 (15)	C21—C20—H20B	108.8
O3—C8—C7	121.67 (15)	C19—C20—H20B	108.8
C9—C8—C7	114.89 (13)	H20A—C20—H20B	107.7
C1—C9—C8	119.57 (15)	O5—C21—C22	120.65 (14)
C1—C9—C10	119.69 (14)	O5—C21—C20	121.30 (15)
C8—C9—C10	120.73 (12)	C22—C21—C20	118.03 (13)
C22—C10—C11	108.70 (11)	C17—C22—C21	118.57 (13)
C22—C10—C9	111.76 (13)	C17—C22—C10	121.92 (13)
C11—C10—C9	111.17 (13)	C21—C22—C10	119.48 (12)
C22—C10—H10A	108.4	C14—C23—H23A	109.5
C11—C10—H10A	108.4	C14—C23—H23B	109.5
C9—C10—H10A	108.4	H23A—C23—H23B	109.5
C16—C11—C12	118.48 (14)	C14—C23—H23C	109.5
C16—C11—C10	122.12 (13)	H23A—C23—H23C	109.5
C12—C11—C10	119.40 (12)	H23B—C23—H23C	109.5
O4—C12—C11	120.52 (15)	C14—C24—H24A	109.5
O4—C12—C13	121.74 (15)	C14—C24—H24B	109.5
C11—C12—C13	117.66 (13)	H24A—C24—H24B	109.5
C12—C13—C14	113.74 (14)	C14—C24—H24C	109.5
C12—C13—H13A	108.8	H24A—C24—H24C	109.5
C14—C13—H13A	108.8	H24B—C24—H24C	109.5
C12—C13—H13B	108.8	C19—C25—H25A	109.5
C14—C13—H13B	108.8	C19—C25—H25B	109.5
H13A—C13—H13B	107.7	H25A—C25—H25B	109.5
C23—C14—C15	108.81 (14)	C19—C25—H25C	109.5
C23—C14—C24	109.51 (15)	H25A—C25—H25C	109.5
C15—C14—C24	110.32 (14)	H25B—C25—H25C	109.5
C23—C14—C13	110.57 (15)	C19—C26—H26A	109.5
C15—C14—C13	107.69 (13)	C19—C26—H26B	109.5
C24—C14—C13	109.92 (14)	H26A—C26—H26B	109.5
C16—C15—C14	113.03 (12)	C19—C26—H26C	109.5
C16—C15—H15A	109.0	H26A—C26—H26C	109.5
C14—C15—H15A	109.0	H26B—C26—H26C	109.5
C16—C15—H15B	109.0		
			
C2—O1—C1—C9	−2.6 (3)	C11—C12—C13—C14	−33.4 (2)
C1—O1—C2—C7	2.8 (2)	C12—C13—C14—C23	173.48 (16)
C1—O1—C2—C3	−177.91 (16)	C12—C13—C14—C15	54.7 (2)
O1—C2—C3—C4	−179.30 (18)	C12—C13—C14—C24	−65.50 (19)
C7—C2—C3—C4	0.0 (3)	C23—C14—C15—C16	−166.89 (15)
C2—C3—C4—F1	179.54 (19)	C24—C14—C15—C16	72.95 (17)
C2—C3—C4—C5	0.2 (3)	C13—C14—C15—C16	−47.01 (19)
F1—C4—C5—C6	−180.0 (2)	C12—C11—C16—O2	−173.90 (13)
C3—C4—C5—C6	−0.7 (4)	C10—C11—C16—O2	6.7 (2)
C4—C5—C6—C7	0.9 (3)	C12—C11—C16—C15	5.1 (2)
O1—C2—C7—C6	179.51 (17)	C10—C11—C16—C15	−174.23 (14)
C3—C2—C7—C6	0.3 (3)	C17—O2—C16—C11	8.7 (2)
O1—C2—C7—C8	−0.4 (3)	C17—O2—C16—C15	−170.42 (12)
C3—C2—C7—C8	−179.60 (17)	C14—C15—C16—C11	19.2 (2)
C5—C6—C7—C2	−0.7 (3)	C14—C15—C16—O2	−161.68 (13)
C5—C6—C7—C8	179.15 (19)	C16—O2—C17—C22	−9.4 (2)
C2—C7—C8—O3	177.60 (17)	C16—O2—C17—C18	170.45 (12)
C6—C7—C8—O3	−2.2 (3)	C22—C17—C18—C19	−21.3 (2)
C2—C7—C8—C9	−2.3 (2)	O2—C17—C18—C19	158.87 (12)
C6—C7—C8—C9	177.86 (17)	C17—C18—C19—C20	47.87 (17)
O1—C1—C9—C8	−0.2 (3)	C17—C18—C19—C25	−72.29 (16)
O1—C1—C9—C10	−179.64 (15)	C17—C18—C19—C26	168.15 (14)
O3—C8—C9—C1	−177.33 (17)	C25—C19—C20—C21	65.87 (19)
C7—C8—C9—C1	2.6 (2)	C26—C19—C20—C21	−173.42 (15)
O3—C8—C9—C10	2.1 (3)	C18—C19—C20—C21	−54.16 (18)
C7—C8—C9—C10	−178.01 (14)	C19—C20—C21—O5	−149.34 (16)
C1—C9—C10—C22	−121.20 (15)	C19—C20—C21—C22	32.2 (2)
C8—C9—C10—C22	59.36 (18)	O2—C17—C22—C21	176.60 (13)
C1—C9—C10—C11	117.14 (16)	C18—C17—C22—C21	−3.2 (2)
C8—C9—C10—C11	−62.29 (18)	O2—C17—C22—C10	−5.4 (2)
C22—C10—C11—C16	−18.99 (19)	C18—C17—C22—C10	174.73 (14)
C9—C10—C11—C16	104.44 (16)	O5—C21—C22—C17	179.38 (15)
C22—C10—C11—C12	161.65 (13)	C20—C21—C22—C17	−2.2 (2)
C9—C10—C11—C12	−74.93 (16)	O5—C21—C22—C10	1.4 (2)
C16—C11—C12—O4	179.18 (16)	C20—C21—C22—C10	179.80 (13)
C10—C11—C12—O4	−1.4 (2)	C11—C10—C22—C17	18.33 (19)
C16—C11—C12—C13	2.2 (2)	C9—C10—C22—C17	−104.74 (16)
C10—C11—C12—C13	−178.44 (15)	C11—C10—C22—C21	−163.73 (13)
O4—C12—C13—C14	149.58 (17)	C9—C10—C22—C21	73.20 (17)

### Hydrogen-bond geometry (Å, °)

**Table:**

*D*—H···*A*	*D*—H	H···*A*	*D*···*A*	*D*—H···*A*
C15—H15B···O4^i^	0.97	2.44	3.3487 (19)	156
C18—H18A···O5^i^	0.97	2.45	3.3821 (19)	162

Symmetry codes: (i) *x*+1, *y*, *z*.
